# Electrophoretic deposition of silk fibroin coatings with pre-defined architecture to facilitate precise control over drug delivery

**DOI:** 10.1016/j.bioactmat.2021.03.046

**Published:** 2021-04-28

**Authors:** Xian Cheng, Dingpei Long, Lili Chen, John A. Jansen, Sander C.G. Leeuwenburgh, Fang Yang

**Affiliations:** a Department of Dentistry-Biomaterials, Radboud Institute for Molecular Life Sciences, Radboud University Medical Center, Philips van Leydenlaan 25, 6525, EX Nijmegen, the Netherlands; b Jiangsu Key Laboratory of Oral Diseases, Nanjing Medical University, Nanjing, 210029, PR China; c Institute for Biomedical Sciences, Center for Diagnostics & Therapeutics, Georgia State University, Atlanta, GA, 30302, USA; d Department of Stomatology, Union Hospital, Tongji Medical College, Huazhong University of Science and Technology, Wuhan, 430022, PR China

**Keywords:** Electrophoretic deposition, Coating, Drug delivery kinetics, Silk fibroin, Nanospheres

## Abstract

The therapeutic precision and clinical applicability of drug-eluting coatings can be substantially improved by facilitating tunable drug delivery. However, the design of coatings which allows for precise control over drug release kinetics is still a major challenge. Here, a double-layered silk fibroin (SF) coating system was constructed by sequential electrophoretic deposition. A mixture of dissolved *Bombyx mori* SF (*bm*SF) molecules and pre-made *bm*SF nanospheres at different ratios was deposited as under-layer. Subsequently, this underlayer was covered by a top-layer comprising *Antheraea pernyi* SF (*ap*SF) molecules (rich in arginylglycylaspartic acid, RGD) to improve the cellular response of the resulting double-layered coatings. Additionally, model drug doxycycline was either pre-mixed with dissolved *bm*SF molecules or pre-loaded into pre-made *bm*SF nanospheres at the same amount before their mixing and deposition. The thickness and nanosphere content of the under-layer architecture were proportional to the deposition time and nanosphere concentration in precursor mixtures, respectively. The surface topography, wettability, degradation rate and adhesion strength were comparable within the double-layered coating system. As expected, RGD-rich *ap*SF top-layer improved cell adhesion, spreading and proliferation compared with *bm*SF top-layer. Furthermore, the amount and duration of drug release increased linearly with increasing nanosphere concentration at fixed deposition time, whereas drug release amount increased linearly with increasing deposition time. These results indicate that the dosage and kinetics of loaded drugs can be quantitatively tailored by altering nanosphere concentration and deposition time as main processing parameters. Overall, this study illustrates the strong potential of pre-defining coating architecture to facilitate control over drug delivery.

## Highlights

• Multi-layered silk fibroin coatings can be achieved by electrophoretic deposition.
• Drugs can be loaded in silk fibroin solution or nanoparticles before deposition.
• Drug release can be easily regulated by the amount of nanoparticles in coatings.
• RGD-rich silk fibroin as a top layer improves cellular attachment and proliferation.

## Graphical abstract

**Figure:**
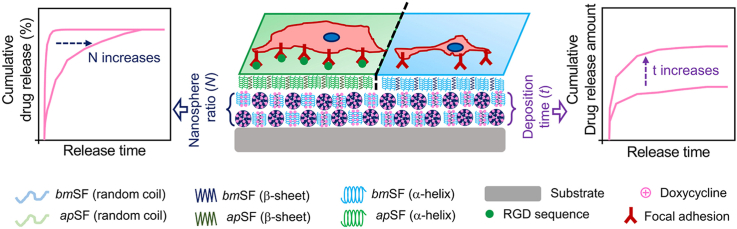

## 1 Introduction

Coatings on medical device surfaces, such as drug-eluting stents [1,2], external fixators [3,4] and implants [5,6], are considered as an effective strategy to act as reservoir for the local release of therapeutic agents. Obviously, the required release duration and dosage of a specific drug can vary among different clinical scenarios. For example, to prevent infection, antibiotic-loaded coatings deposited on orthopedic fixation devices for open fracture patients usually require sustained drug delivery at high dosages [7,8]. However, antibiotic delivery at insufficient amounts and/or duration may result in clinical complications such as limited prophylactic efficacy or antibiotic resistance [8]. Therefore, precise control over the amount and kinetics of local antibiotic delivery will improve therapeutic precision and expand the clinical applicability of drug-eluting coatings [9].

Electrophoretic deposition (EPD) is one of the most attractive techniques to produce coatings for biomedical applications due to advantages such as: i) deposition in mild aqueous environments at room temperature [10]; ii) fabrication of homogeneous and conformal coatings on complex or porous medical devices [11]; iii) tight control over coating properties via processing parameters [12]; and iv) compatibility for industrial upscaling due to the relatively simple equipment and short fabrication time [13]. Previously, drugs (e.g. antibiotics) have been mixed with EPD coating precursors before their co-deposition [11,12] or post-loaded via adsorption [14]. However, these drug loading strategies offer poor control over drug delivery. More recently, several approaches have been employed to overcome this limitation, such as regulation of the degradation rate of the coating matrix [15], cross-linking of the drug with the coating matrix [16], and adding drug delivery microcarriers into the coating matrix [6,13,17,18]. However, these modified coating systems still suffer from several shortcomings. For example, excessively fast coating degradation may compromise the cytocompatibility of the coating [15,19]. Moreover, the use of an additional crosslinker [16] or coating components (e.g. metal-organic frameworks [6], carbon nanotube [13], and graphene oxide sheets [18]) may raise concerns regarding biosafety and manufacturing costs.

Therefore, we and other groups reported that silk fibroin (SF) coatings can be deposited in a simple and straightforward manner by using dissolved *Bombyx mori* SF (*bm*SF) molecules [11,12,[20], [21], [22]] or pre-made *bm*SF nanospheres as precursor components [23]. The EPD coating comprising *bm*SF nanospheres was able to decelerate the release of entrapped drugs considerably as compared to monolithic coatings composed of *bm*SF molecules [23]. Nevertheless, drug release kinetics could not yet be tuned to desire. In the current study, we aimed to proceed with the optimization of EPD-deposited SF coatings and hypothesized that tunable drug release kinetics can be obtained by depositing coatings composed of both *bm*SF molecules and *bm*SF nanospheres at different ratios.

*bm*SF has recently attracted attention for biomacromolecule delivery due to its stabilizing effect on sensitive biological compounds (e.g. antibiotics) [24] and its hypoallergenicity [25,26]. However, *bm*SF molecules do not contain cell recognition motifs, such as arginylglycylaspartic acid (RGD), which facilitates cell adhesion and spreading. On the other hand, *Antheraea pernyi* SF (*ap*SF), an alternative silk protein, is rich in such RGD sequences. In view of the high costs of *ap*SF, we propose the deposition of *ap*SF molecules as top-layer onto a *bm*SF under-layer as a cost-effective way to favor the cellular response to an SF-coated device without comprising the beneficial properties of *bm*SF.

In this study, a novel double-layered SF coating system was prepared by sequential EPD. First, a mixture of dissolved *bm*SF molecules and pre-made *bm*SF nanospheres at different ratios was deposited as under-layer to enable tunable drug delivery. Subsequently, a top-layer comprising *ap*SF molecules was deposited to further improve the cellular response to the coating. To investigate the deposition mechanism of coatings, the colloidal stability of EPD solutions/suspensions was studied as a function of pH. Subsequently, we studied the influence of deposition time and concentration of nanospheres in precursor mixtures on the thickness and nanosphere content of the under-layer architectures. Moreover, the surface topography, wettability, degradation, adhesion strength and cytocompatibility of the coating system were characterized. Furthermore, doxycycline, a broad-spectrum antibiotic, was selected as model drug to investigate the relationship between coating processing parameters, coating architecture and drug delivery performance.

## 2 Materials and methods

### 2.1 Preparation of *bm*SF_sol_, *ap*SF_sol_, and *bm*SF_sus_

Aqueous solutions of *bm*SF (*bm*SF_sol_) or *ap*SF molecules (*ap*SF_sol_) were prepared as previously reported [11,27]. Briefly, *Bombyx mori* and *Antheraea pernyi* silk cocoons, both provided by the State Key Laboratory of Silkworm Genome Biology of Southwest University, China, were first boiled in 0.02 M Na_2_CO_3_ aqueous solution for 30 min and washed with water for 1 h to remove sericin. After drying, the degummed *Bombyx mori* silk fibers were dissolved in 9.3 M LiBr aqueous solution at 1:4 (w/v) bath ratio for 4 h at 60 °C [11]. The degummed *Antheraea pernyi* silk fibers were dissolved in melted Ca(NO3)_2_·4H_2_O at bath ratio w/v: 1:10 at 105 °C for 5 h [27]. Subsequently, the protein solutions were dialyzed against water using a 3500 Da cut-off dialysis membrane for 72 h to remove salts. Insoluble residues were removed by centrifugation at 5000 rpm (Universal 32R, Hettich, Germany) for 1h. The final protein concentration in *bm*SF_sol_ and *ap*SF_sol_ was 8 wt % and 2.5 wt% measured by dry weighing, respectively.

The aqueous suspension of *bm*SF nanospheres (*bm*SF_sus_) was prepared using a previously described precipitation approach [23]. To this end, *bm*SF_sol_ (5 wt %) was added dropwise into acetone to form *bm*SF nanospheres. The *bm*SF nanospheres were washed with water and centrifuged at 10000 rpm (9391 rcf), (Centrifuge 5415r, Eppendorf, Germany) for three times to remove acetone. A sonicator (UP50H, Hielscher, Germany) was used to ultrasonically disperse *bm*SF_sus_ in an ice bath for 30 s (50% amplitude) before use.

### 2.2 Drug pre-loading into *bm*SF nanospheres in *bm*SF_sus_ and pre-mixing drug with *bm*SF molecules in *bm*SF_sol_

The model drug doxycycline hyclate (D9891, Sigma, Germany) was either pre-loaded into *bm*SF nanospheres (*bm*SF_sus_) or pre-mixed with *bm*SF molecules (*bm*SF_sol_) at the same amount.

To obtain drug loaded *bm*SF nanospheres, doxycycline solution was dropped into *bm*SF_sol_ (5 wt %) under stirring (100 rpm) at different doxycycline/SF weight ratios of 0.05, 0.10, 0.15, 0.20, 0.25 or 0.30. Then, the various solutions were added dropwise into acetone to precipitate doxycycline-loaded *bm*SF nanospheres [23]. Acetone did not to compromise the pharmaceutical activity of doxycycline [28,29]. These drug-loaded nanospheres were washed with water and centrifuged at 10000 rpm (9391 rcf) for 5 min for three times to remove any residual acetone and unloaded doxycycline.

High performance liquid chromatography (HPLC, BioTek, synergy HTX, USA) at 360 nm was used to measure the amount of unbound doxycycline in the washed supernatants with a LiChrospher RP-18 end capped HPLC column (125 mm × 4 mm, particle size 5 μm), as previously described [28]. The loading capacity and encapsulation efficiency of doxycycline for *bm*SF nanospheres were calculated using the following formulae (n = 3):

$$\text{Loading capacity \% }=\;\frac{\text{total amount of drug }-\text{ amount of unbound drug}}{\text{weight of bmSF nanospheres}}\times 100\%$$

$$\text{Encapsulation efficiency }\left(\text{\%}\right)\;=\;\frac{\text{total amount of drug }-\text{ amount of unbound drug}}{\text{total amount of drug}}\times 100\%$$

The weight ratio (doxycycline/SF) of drug added into *bm*SF_sol_ to reach optimum doxycycline encapsulation efficiency was used for preparation of drug-loaded nanospheres in all further assays.

To pre-mix doxycycline with *bm*SF molecules in *bm*SF_sol_ (1 wt %), doxycycline solution was dropped into *bm*SF_sol_ to reach a concentration (doxycycline/SF) of 3.38% comparable to the drug loading capacity of *bm*SF nanospheres after optimization. The mixture solution was stirred at 100 rpm for 24 h before use.

### 2.3 Characterization of *bm*SF_sol_, *ap*SF_sol_, and *bm*SF_sus_

To assess the morphology of the *bm*SF nanospheres, samples were freeze-dried at −52.1C° for 3 days (VirTis Benchtop Pro, SP Industries, Inc., USA) and examined by scanning electron microscopy (SEM, Sigma-300, Zeiss, Germany) after sputtering 10 nm chromium coating. Dynamic light scattering (Zetasizer, Nano-S, Malvern Instruments, U.K.) was applied to test the ζ-potential of samples (n = 3). The pH of samples was measured using a pH meter (PHM210, Hach, U.S.A.).

### 2.4 EPD of coatings from *bm*SF_sol+sus_ and *ap*SF_sol_

Pure titanium disks (grade 2, Baoji Titanium Industry, China) were used as sample substrates. Grit 600 and Grit 2500 grinding sandpapers (Struers, the Netherlands) were used sequentially to smoothen the titanium surface. Subsequently, the titanium disks were washed in acetone, ethanol, and water in an ultrasonic bath for 10 min in succession, followed by argon plasma treatment (radio-frequency glow discharge machine, Harrick, U.S.A.) for 10 min.

Different amounts of *bm*SF_sol_ (1 wt %) and *bm*SF_sus_ (1 wt %) without and with drug were mixed together to obtain a series of *bm*SF_sol+sus_. The total SF concentration was fixed at 1 wt %, while the relative amount of nanospheres was varied between 0, 25, 50, 75 and 100%. These experimental groups without drug were designated as SFN0, SFN25, SFN50, SFN75 and SFN100, respectively, whereas groups with drug were designated as SFND0, SFND25, SFND50, SFND75, and SFND100, respectively (Table 1).

**Table 1:** Abbreviation and composition of experimental groups.

Group	*bm*SF_sus/_*bm*SF_sol_ (%/%)	Total *bm*SF concentration (wt %)	Total drug concentration (doxycycline/*bm*SF) (w/w %)
SFN0	0/100	1	0
SFN25	25/75	1	0
SFN50	50/50	1	0
SFN75	75/25	1	0
SFN100	100/0	1	0
SFND0	0/100	1	3.38
SFND25	25/75	1	3.38
SFND50	50/50	1	3.38
SFND75	75/25	1	3.38
SFND100	100/0	1	3.38

To allow for coating deposition using EPD, one titanium disk was used as the working positive electrode, and another titanium disk with the same size was applied as the counter electrode. The distance between the two electrodes was set at 1 cm. The EPD process was conducted using a direct current power supply (Model 6614C, Agilent, U.S.A.) at a constant electric field of 5 V/cm. The *bm*SF underlayers were first deposited from various types of *bm*SF_sol+sus_ mixtures_._ Subsequently, these *bm*SF underlayers were covered by an *ap*SF top-layer by depositing *ap*SF_sol_ (1 wt %). For each deposition, a freshly prepared 10 mL *bm*SF_sol+sus_ or *ap*SF_sol_ was used and mildly stirred on a magnetic stirrer (50 rpm) during deposition. After deposition, the coated disks were gently rinsed with water and slowly air-dried in a box to prevent cracking [23]. For drug release and cell culture studies, the coating made on the bottom side of disk which touched the box during air drying was removed by a Grit 600 grinding sandpaper (Struers, the Netherlands).

### 2.5 Characterization of the coating system

SEM was employed to examine the surface topography of the coating under-layer and top-layer after coverage by a 10 nm chromium layer.

Fourier transform infrared spectroscopy (FTIR, Spectrum two with the UATR accessory, PerkinElmer, the Netherlands) was used to analyze the molecular conformation of the coated *bm*SF with and without doxycycline loading. To calculate the β-sheet and α-helix content of the *bm*SF under-layer with different microstructures, the contribution of the different SF conformations to the amide I region (1595-1705 cm-1) was determined by Fourier self-deconvolution using PerkinElmer software and subsequent curve fitting by OriginPro software according to a previously reported method (n = 3) [30].

A profilometer (Proscan 2100, Scantron Industrial Products, U.K.) was used to measure the thickness and roughness of the coatings (n = 3). The water contact angle was measured using an Optical Tensiometer (Theta Lite, Biolin Scientific, Sweden) to assess the wettability of the coating surface (n = 3).

To determine the degradation kinetics of the coatings, a degradation test was performed in PBS solution at a shaking rate of 90 rpm at 37 °C for 14 days. PBS solution was refreshed every 24 h. At specified time points, samples were taken out, gently rinsed with water, dried in an oven, and weighted (n = 3).

A lap shear tensile test was performed to measure the adhesion strength, as previously described (Supporting information, Fig. S1a) [31]. Briefly, a coated titanium substrate was glued at its coated side to an uncoated titanium substrate using an instant epoxy adhesive (Loctite 415, Loctite, USA). The substrates were vertically fixed on a Universal Testing Machine (858 mini bionix II, MTS Systems, U.S.A.). The tensile test was carried out using a constant crosshead displacement of 0.50 mm per min until failure occurred, as was evidenced by a sudden drop in load. The samples (Supporting information, Fig. S1b) were inspected using a stereoscopic microscope (MZ12, LEICA, Germany) to ensure that failure occurred at the coating-substrate interface rather than the coating-adhesive interface.

### 2.6 Measurement of drug release profiles from the coating system

The disks (diameter of 14.5 mm; thickness of 1 mm) with different drug loaded coatings were put in 24 well plates and immersed in 1 mL PBS solution to ensure the sink conditions were maintained during the release test. The plates were sealed with adhesive sealing film (SecureSeal, Simport, Canada) and shaken with a rate of 90 rpm at 37 °C for 14 days to perform a drug release study. At specific time points (1 h, 2 h, 4 h, 8 h, 12 h, 1 d, 2 d, 3 d, 4 d, 7 d, 11 d, 14 d, and 17 d), the supernatant was collected and refreshed with new PBS solution. The concentrations of doxycycline in collected supernatants were measured using HPLC (n = 3).

We further fitted the drug release data to three commonly-used diffusion-controlled models, i.e. the first-order (Equation (1)), Higuchi (Equation (2)), Korsmeyer-Peppas (Equation (3)):

(1) -In(1-Q)=aT+b

(2) $${\text{Q}=\text{cT}}^{0.5}+\text{d}$$

(3) $${\text{Q}=\text{kT}}^{\text{n}}$$

where Q is the accumulative drug released % at the specific release time *T*; a, *b*, *c*, *d, n,* and *k* are the constants.

### 2.7 Cell culture

NIH3T3 cells (mouse embryonic fibroblastic cell line) obtained from American Type Culture Collection were applied to study the influence of *ap*SF top-layer on cell adhesion, spreading and proliferation behavior. SFN50 coating was applied as experimental group. We used the coating with the same under-layer of SFN50, but a different top-layer deposited from *bm*SF_sol_ instead of *ap*SF_sol_ as the control group, which was designated as SFN50_b_ (Table 1). The culture medium was Dulbecco's modified essential medium (Gibco, Invitrogen, Scotland) supplemented with 10% calf serum (Gibco, Invitrogen, Scotland) and 1% penicillin/streptomycin. Cells were seeded on UV-light sterilized samples at a density of 5000 cells·cm^−2^.

### 2.8 Immunofluorescent staining and analysis

For immunofluorescent observation, cells were fixed at 24 h after cell seeding using 4% paraformaldehyde for 10 min. Then, they were permeated using 0.1% Triton X-100 for 10 min, and blocked with 1% BSA for 1h. To stain the focal adhesions, samples were incubated with anti-vinculin primary antibody (1:400, ab129002, Abcam, U.S.A.) at 4 °C overnight, followed by incubation with Alexa-Fluor 647-conjugated secondary antibody (1:400, ab150083, Abcam, U.S.A.) at room temperature for 1h. Stained samples were observed using a fluorescence microscope (Axio Imager Microscope Z1, Zeiss, Germany). Images analysis was performed by Image J (NIH, USA). The quantitative measurement of focal adhesion area per cell followed a step-by-step protocol [32]. To label the F-actin, samples were incubated with TRITC-phalloidin (1:1000, P1951, Sigma, U.S.A.) for 30 min. Image J was used to trace the cell borders to quantify the cell spreading area. At least 30 cells were analyzed per group.

Similarly, to stain Ki-67, a marker of proliferation present during all active phases of the cell cycle (G1, S, G2, and mitosis) [19], samples first were incubated with anti-Ki67 antibody (1:400, ab16667, Abcam, U.S.A.) and then with Alexa-Fluor 594-conjugated secondary antibody (1:500, A32740, Abcam, U.S.A.). To label the nucleus, cells were incubated DAPI (1:2000, D9542, Sigma, U.S.A.) for 15 min. The positive Ki-67 cell % were calculated based on the images taken from three random fields for each sample and three samples were analyzed per group (n = 3).

### 2.9 Cell adhesion

After incubating the samples for 2 and 4 h, they were taken out, rinsed with PBS to remove any non-adherent cells, and transferred to a new plate. The adherent cell number was measured by QuantiFluor dsDNA System kit (Promega Corporation, Madison, USA) using spectrophotometry according to the manufacturer's instructions. The cell adhesion rate was counted as the ratio between the adherent cell number at 2 and 4 h and initial cell seeding number (n = 3).

### 2.10 Cell proliferation

Cell counting kit-8 (CCK-8, Dojindo, Japan) was applied at the specific time points (1, 3, and 7 d) to measure the total cell number (n = 3). The CCK-8 result was tested spectrophotometrically (Bio-Tek FL600 microplate fluorescence reader, Biotek, U.S.A.) according to the manufacturer instructions.

### 2.11 Cell cytocompatibility

After 24h of incubation, Lactate Dehydrogenase (LDH) Assay Kit (Thermo Fisher Scientific, U.S.A.) was used for determining cytocompatibility of drug-loaded coatings by measuring the LDH activity released from damaged cells. cell culture coverslips without and with 5% DMSO were used as positive and negative controls, respectively, while wells added with 2% (v/v) Triton-X100 and only culture medium were used as the high and low controls, respectively. The results from the LDH assay were tested spectrophotometrically according to the manufacturer instructions. Three samples per group were tested. The cytocompatibility was calculated using the formula as previously reported [33]:

$$\text{Cytocompatibility }\left(\text{\%}\right)\;=\;\left(1-\frac{\text{experimental value}-\text{ low control}}{\text{high control}-\text{low control}}\right)\times 100\text{\%}$$

### 2.12 Statistical analysis

All quantitative data were expressed as mean ± standard deviation (SD). Statistical analyses were performed using GraphPad Prism 7.0 One-way ANOVA followed by Tukey post hoc test was used for multiple comparisons.

## 3 Results and discussion

### 3.1 Electrophoretic assembly mechanism of the coatings

**Fig. 1:**
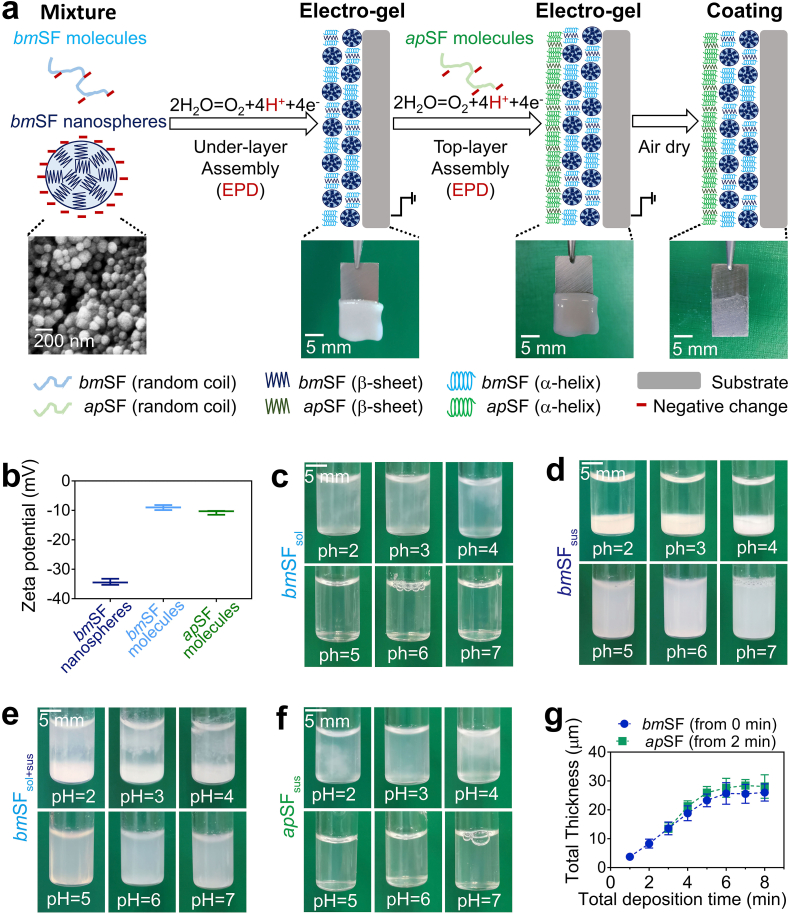
**EPD process and mechanism of the coating system. (a)** Schematic illustration of the coating system involving sequential EPD of a mixture of dissolved *bm*SF molecules and pre-made *bm*SF nanospheres as under-layer and *ap*SF molecules as top-layer. (**b)** ζ-potential values. Photos showing colloidal stability of **(c)***bm*SF_sol_, **(d)***bm*SF_sus_, **(e)***bm*SF_sol+sus_ of SFN 50 group, or **(f)***ap*SF_sol_ as a function of pH. **(g**) Coating thickness of SFN 50 as a function of total deposition time. The *ap*SF top-layer is deposited after a 2 min deposition of *bm*SF under-layer. Error bars represent standard deviations.

The spherical morphology of *bm*SF nanospheres was confirmed by SEM (Fig. 1a). The pH of *bm*SF_sus_ (1 wt %), *bm*SF_sol_ (1 wt %), and *ap*SF_sol_ (1 wt %) was about 7.5, and their ζ-potential values at pH 7.5 were −34.3, −9.0, −10.7, respectively (Fig. 1b). The measured ζ-potential values were consistent with previous studies [12,34,35], indicating that *bm*SF nanospheres, *bm*SF molecules, and *ap*SF molecules were all electronegative. The higher negative charge of *bm*SF nanoparticles compared to *bm*SF molecules may be due to that the negatively charged N-termini are exposed at the surface whereas the positively charged C-termini self-assembled inside the nanoparticles at a neutral condition [36].

Previous work demonstrated that *bm*SF molecules [11,12,[20], [21], [22]] or nanospheres [23] can be electrophoretically deposited onto substrates in an aqueous environment. When applying a voltage, negatively charged *bm*SF molecules or nanospheres move towards the anode substrate, where water is oxidized, causing pH reduction and neutralizing the negative surface charge of *bm*SF molecules or nanospheres [11,23]. The corresponding weakening of repulsive interactions between *bm*SF molecules or nanospheres induces their irreversible deposition to form an electro-gel on the substrate, which finally transforms into a coating after drying [11,23].

Here, we chose SFN50 as experimental group to investigate the mechanism and kinetics of depositing *bm*SF_sol+sus_. The colloidal stability of *bm*SF_sol+sus_ as a function of the pH was studied as compared to the *bm*SF_sol_ or *bm*SF_sus_. At pH ≥ 5, the dispersion of the *bm*SF_sol+sus_ (Fig. 1e), similar to the *bm*SF_sol_ (Fig. 1c) and *bm*SF_sus_ (Fig. 1d) was stable. However, when the pH was reduced below 4, flocculation (Fig. 1c) and precipitation (Fig. 1d) occurred in solutions and suspensions, respectively, which coincided with the reported pI of *bm*SF (~4.2) [22]. The same phenomena were observed in *bm*SF_sol+sus_ (Fig. 1e), indicating that *bm*SF molecules and nanospheres in *bm*SF_sol+sus_ could be co-deposited onto the substrate (Fig. 1 a). Moreover, the *ap*SF_sol_ showed the same behavior regarding dispersion stability at different pH values as the *bm*SF_sol_ (Fig. 1f *v.s*c). This is likely due to the comparable pI values of *ap*SF (~4.3) [35] and *bm*SF (~4.2) [22], which indicates that the EPD mechanism of *ap*SF molecules is similar to *bm*SF molecules (Fig. 1 a).

The key processing parameters determining EPD coating thickness have been summarized by Besra, and include the electric field, deposition time and suspension characteristics [37]. Our previous study demonstrated that an electric field of 5V/cm and protein concentration of 1.0 wt % enabled deposition of homogeneous coatings from *bm*SF_sol_ [11] or *bm*SF_sus_ [23]. Therefore, we also applied 5V/cm and 1.0 wt % for depositing our coatings. Our data indicated that during deposition of the *bm*SF under-layer, its thickness increased linearly (R^2^ = 0.993) at 4.5 μm/min when the deposition time increased from 0 to 6 min (Fig. 1g; Supporting information, Fig. S2a). Consequently, a moderate time of 2 min was chosen for under-layer deposition before top-layer deposition. The thickness of top-layer increased linearly during deposition at 5.1 μm/min (R^2^ = 0.960) with increasing deposition time from 0 to 4 min (Fig. 1g; Supporting information, Fig. S2b).

These results indicate that the EPD process of both layers is Faradaic [31], and follows the classical Hamaker equation, which predicts a linear increase on deposited mass (thickness) with the increase of deposition time at a given electric field and protein concentration [23]. However, when the time exceeded 6 min, the increase in coating thickness slowed down due to self-limitation. Water was oxidized at the interface between substrate surface/electrolyte, whereas deposition took place at the interface between the electro-gel surface and electrolyte (Fig. 1a). With increasing time, the increasing distance between these two interfaces induced the self-limitation phenomena that reduced the growth kinetics of the coating [23,31].

### 3.2 Material characterization of the coating system

**Fig. 2:**
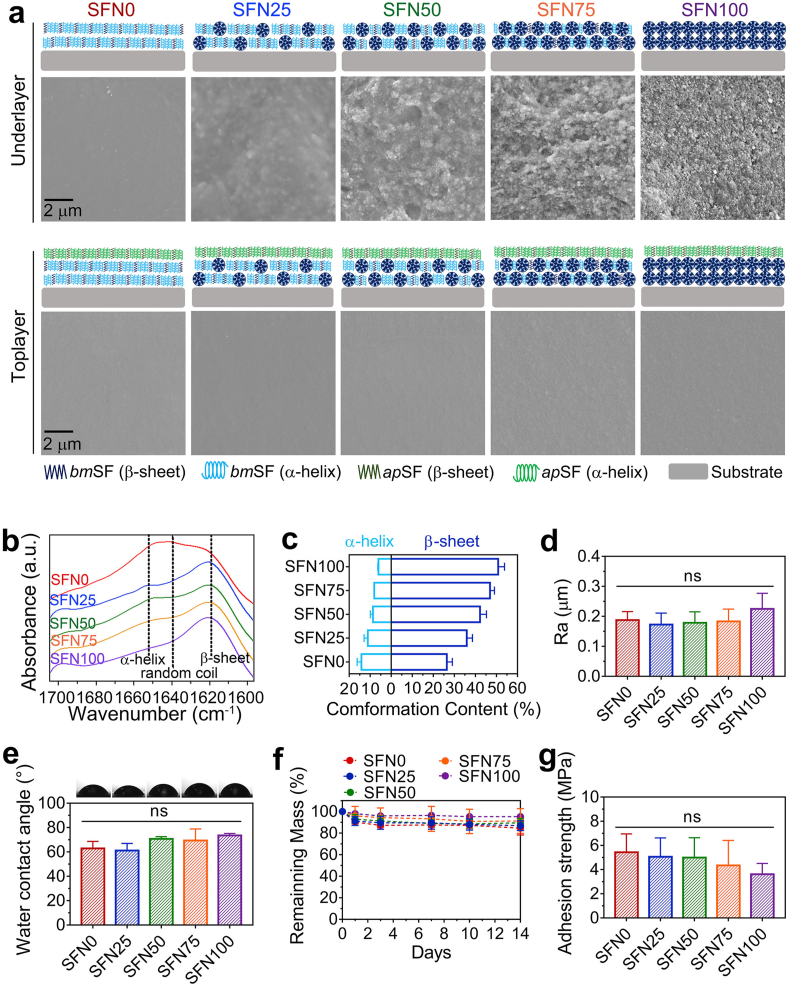
**Material characterization of the coating system. (a)** SEM images of under-layer and top-layer surfaces of the coating system. (**b**) FTIR measurement on under-layer surfaces showing the absorbance spectra of the amide I region (between 1695 and 1595 cm–1) of *bm*SF molecules. (**c)** Conformation contents of *bm*SF molecules calculated by Fourier self-deconvolution from the amide I region spectra. (**d)** Surface roughness of the coating system. (**e)** Surface wettability of the coating system determined by water contact angle measurements and representative images of water droplets. (**f)** Remaining mass of coatings immersed in PBS after 1, 3, 7, and 14 days. (**g)** Adhesion strength of the coating system measured by lap shear tensile testing. Error bars represent standard deviations (ns: no significance).

Coating deposition times of the under-layer and top-layer were fixed at 2 and 1 min, respectively, to study the physicochemical properties of coatings with different under-layer architectures (Fig. 2a). The thicknesses of these coatings (~13 μm) were comparable (Supporting information, Fig. S3). To investigate if the nanosphere concentration in *bm*SF_sol+sus_ correlated with the nanosphere content of under-layer architectures, SEM and FTIR were used to examine the *bm*SF under-layer surface without depositing an *ap*SF top-layer.

With increasing nanosphere concentration, SEM images showed that the underlayer surface became more irregular, as more *bm*SF nanospheres were observed embedded in the coating architectures (Fig. 2a). The SF conformation of different coatings was calculated by Fourier self-deconvolution from the spectra of the amide I region as measured by FTIR (Fig. 2b and c). Previous work indicated that deposition of *bm*SF_sol_ induced an obvious conformational transformation from random-coil to α-helical state of *bm*SF [21]. In contrast, the formation of *bm*SF nanospheres led to a large amount of β-sheet conformation [34], which was not affected by the EPD process [23]. With increasing nanosphere concentration, the β-sheet and α-helical contents in deposited coatings linearly increased (R^2^ = 0.967) and decreased (R^2^ = 0.955), respectively (Fig. 2c; Supporting information, Fig. S4). These results indicate that the nanosphere content of the under-layer architectures correlates linearly with the nanosphere concentration in *bm*SF_sol+sus_*.*

Subsequently, we characterized the top-layer of coatings. SEM images showed that the application of *ap*SF top-layers flattened the underlying under-layers (Fig. 2a), resulting in a comparable surface roughness (Fig. 2d). The water contact angles did not differ significantly among the various experimental groups (Fig. 2e), since the chemical composition and surface topography of the top-layer were similar [38,39]. The smooth surface and moderate hydrophobicity of our coatings were similar to percutaneous parts of commercially available titanium implants [39].

The deposited coatings showed very limited degradation within 14 days (Fig. 2f), which was in line with a previous study on SF-based materials [23]. The adhesion strength between coating system and substrate was investigated in a lap shear tensile test (Fig. 2g and Fig. S1, Supporting information). The various under-layers adhered equally strong to the substrate. The adhesion strength (3.6–5.4 MPa) of the coating system was similar to the strength of other reported EPD-deposited polymer-based coatings (1.5–8 MPa) [[10], [11], [12],^16^,^40^], but lower than the adhesion strength of *bm*SF coatings observed previously (6.7–8.2 MPa) [23]. This reduced adhesion strength can be attributed to increased coating thickness caused by the double-layer design. However, the adhesion strength of our coating system is sufficient for devices that are not subjected to high shear and tensile forces, such as the transcutaneous part of percutaneous implants [17].

### 3.3 *ap*SF top-layer improves initial cell response of the coatings

**Fig. 3:**
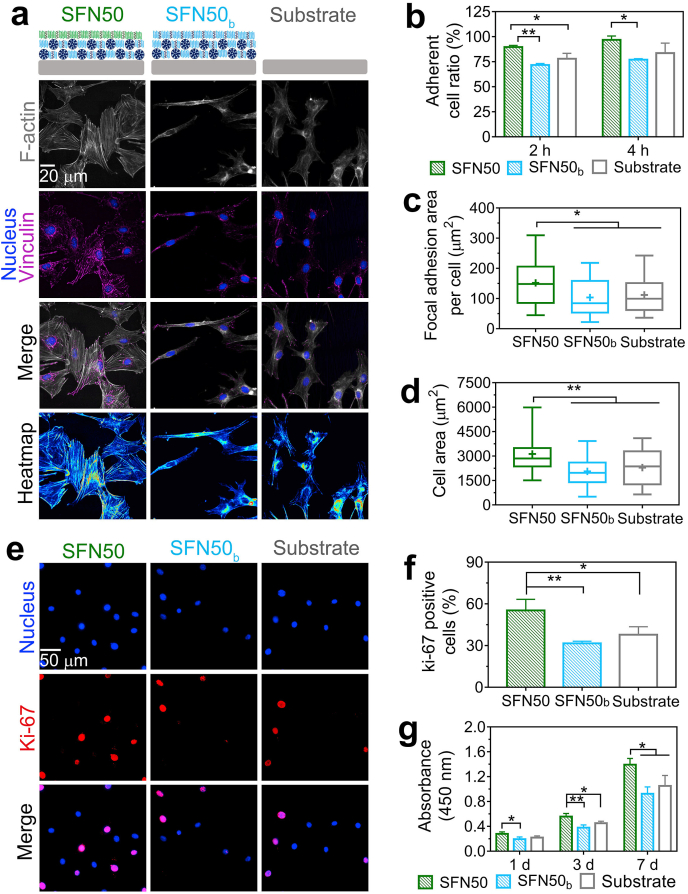
**apSF top-layer improves initial cell response of the coatings. (a)** Cell spreading at 24 h shown by immunofluorescent images of vinculin (purple), F-actin (grey), and nucleus (blue), as well as the corresponding heatmap of F-actin. (**b**) Adherent cell ratio of cells cultured on different surfaces at 2 and 4 h measured by DNA content assay. (**c)** Quantitative analysis of focal adhesion area per cell. (**d)** Quantitative analysis of cell area. (**e**) Cell proliferating phase at 24h shown as immunofluorescent images of nucleus (blue) and Ki-67 protein (red). **(f)** Quantitative analysis of the positive ki-67 cells %. (**g)** Cell number at 1, 3, and 7 d measured by CCK-8. Error bars represent standard deviations (*: p < 0.05 and **: p < 0.01). (For interpretation of the references to colour in this figure legend, the reader is referred to the Web version of this article.)

To evaluate the effect of *ap*SF top-layer on cell behavior, initial cell response including adhesion, spreading and proliferation was assessed for different material surfaces, i.e. *ap*SF top-layer, *bm*SF top-layer and uncoated substrate (Fig. 3a). Before seeding cells, the samples were sterilized by UV-light in this study. However, other routine sterilization methods, such as gamma irradiation, can also be used to sterilize SF materials [41] and loaded doxycycline [42].

After 2 h of cell seeding, the adherent cell ratio was higher on *ap*SF top-layer than on *bm*SF top-layer (p < 0.01) and uncoated titanium substrate (p < 0.05) (Fig. 3b). After 4 h, the number of adherent cells increased for all different material surfaces, but the adherent cell ratio on *bm*SF top-layer was still significantly lower than on *ap*SF top-layer (p < 0.05) (Fig. 3b). This result indicates that an *ap*SF top-layer accelerates the cell adhesion process as compared to *bm*SF top-layer and titanium substrate. The faster cell adhesion on a material surface with a high content of RGD sequences has also been observed elsewhere [19,43,44]. Furthermore, a previous study found that the incorporation of recombinant RGD motif-containing peptides derived from *apSF* into *bm*SF films was even more effective for rapid cell adhesion of L929 cells (another murine fibroblast cell line) than incorporation of chemically synthesized RGD peptides [43].

After 24 h, cells accomplished their adhesion and spreading process [19]. The immunostaining images of F-actin and subsequent analysis show that cells on *apSF* top-layer spread over a larger area (Fig. 3a and d) (p < 0.01) with more abundant cytoskeleton organization (Fig. 3a) than cells on the other substrates. The immunostaining images of vinculin and subsequent corresponding analysis (Fig. 3a and c) display that more vinculin-associated focal adhesions were formed in cells on *apSF* top-layer as compared to those on *bm*SF top-layer and titanium substrate (p < 0.05). Previous studies also reported that a high content of RGD sequences in materials upregulated vinculin expression and facilitated focal adhesion formation [19,45], which coincides with our results. Via linking F-actin to the vinculin at focal adhesion, vinculin can trigger a series of phosphorylation events to promote cytoskeleton organization and cell spreading [46].

To study proliferation during the cell cycle, Ki-67 was stained after 24 h of cell culture. Ki-67 is a marker of proliferation present during all active phases of the cell cycle (G1, S, G2, and mitosis) [19]. Our results (Fig. 3e and f) showed that the proportion of Ki-67 positive cells on *ap*SF top-layer was higher than on *bm*SF top-layer (p < 0.01) and uncoated titanium substrates (p < 0.05). This indicates that the *ap*SF top-layer stimulated more cells to enter the active phases of the cell cycle for proliferation (i.e. proliferative phase). Subsequently, CCK-8 tests were performed to evaluate cell growth from 1 to 7 days (Fig. 3g). A significantly higher number of cells was observed at all time points on top of *ap*SF top-layer as compared to other experimental groups. This observation can be attributed to the faster cell adhesion, better cell spreading and higher proportion of cells entering the proliferative phase on *ap*SF surface as compared with the other material surfaces. Additionally, a slightly lower proliferative activity of cells was observed on SFN50b as compared to uncoated titanium substrate although there was no statistic significance. This may be attributed to the relatively weak cell adhesion to *bm*SF due to the absence of cell recognition motifs and its negatively charged nature [47].

### 3.4 Tunable drug delivery of the coating system

**Fig. 4:**
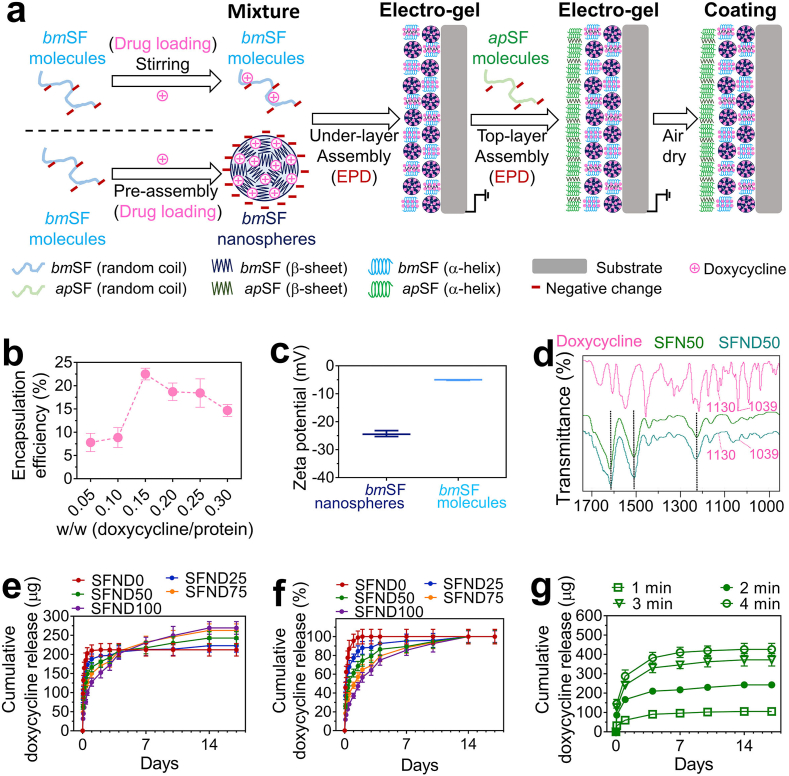
**Drug loading and release of the coating system.** (**a)** Schematic illustration of the process of drug loading onto the coatings. Model drug, doxycycline, was either pre-mixed with dissolved *bm*SF molecules or pre-loaded into pre-made *bm*SF nanospheres before under-layer deposition. (**b)** Encapsulation efficiency of *bm*SF nanospheres as a function of the weight ratio (drug/protein). (**c)** ζ-potential values of drug-loaded *bm*SF nanospheres and molecules. (**d)** FTIR measurement on pure doxycycline powder, underlayer surface of SFN50 and underlayer surface of SFND50. Drug release profiles of the coating system shown as the (**e)** cumulative release amount or (**f)** cumulative release percentage of the maximum release amount using different nanosphere concentrations and the same under-layer deposition time (2 min). (**g)** Drug release profiles of SFND50 group for different under-layer deposition times at a fixed nanosphere concentration (50%). Error bars represent standard deviations.

The model drug doxycycline was either pre-loaded into *bm*SF nanospheres in *bm*SF_sus_ and pre-mixed with *bm*SF molecules in *bm*SF_sol_ at the same amount before their mixing and deposition (Fig. 4a). The optimum weight ratio (doxycycline/SF) of doxycycline added into *bm*SF_sol_ when preparing drug loaded *bm*SF nanospheres was 0.15, where the maximum drug encapsulation efficiency was 22.5 ± 1.3% (Fig. 4b) and the loading content was 3.38 ± 0.19%. Compared to the encapsulation efficiency of some lipophilic drugs, e.g. curcumin (48 ± 2.7%), the one of doxycycline was relatively low [48]. This indicates that part of the water-soluble doxycycline stayed in the aqueous phase and could not co-precipitate with SF. Also, the washing steps may lead to drug loss. Future studies should be done to improve the drug encapsulation efficiency.

Drug loading decreased the negative surface charge of nanospheres from −34.3 to −25.7 mV (Figs. 1b and 4c). This phenomenon results from partial neutralization of negatively charged *bm*SF nanospheres by positively charged doxycycline [34], and indicates that electrostatic interactions may form between the doxycycline and *bm*SF [17]. On the other hand, the zeta potential value of *bm*SF_sol_ decreased from −9.0 to −5.1 mV after pre-mixing drug with *bm*SF molecules in *bm*SF_sol_ (Figs. 1b and 4c), indicating that polyelectrolyte complex may form between doxycycline and *bm*SF molecules [12].

FTIR was performed on the under-layer surface without a top-layer (SFND50 as an example group) (Fig. 4d). The SFND50 coating possessed three typical absorbance peaks at 1619 cm^−1^ (amide I band), 1512 cm^−1^ (amide II band), and 1230 cm^−1^ (amide III band) for silk protein [30]. Extra absorbance peaks at 1309 and 1130 cm^−1^ for doxycycline [28] were also found in the spectrum of the SFND50 coating as compared to the SFN50 coating without drug, indicating successful loading of doxycycline.

HPLC was applied to monitor drug release profiles from the coating system. After the first 12 h, all formulations of the coating system displayed an initial burst release, even though the burst release rate was reduced from 87 to 27 % by increasing the nanosphere concentration from 0 to 100 % (Supporting information, Fig. S6). For antibiotic release coatings, an initial burst release of high concentration of antibiotics is generally considered required to eradicate bacterial contamination upon surgery, whereas a long-term sustained local release of antibiotics can prevent bacterial re-colonization on implant surfaces [14,17,29]. Our and other studies have demonstrated that antibiotics released from *bm*SF molecule EPD coating [12] or *bm*SF nanosphere EPD coating [23] can retain their drug effectiveness. These findings point to a stabilizing effect of *bm*SF molecules on sensitive biological compounds (e.g. antibiotics) upon incorporation into *bm*SF matrices [24]. Besides, the drug-loaded coating (SFND50 as an example group) was not cytotoxic to fibroblasts (Supporting information, Fig. S7).

Applying the same under-layer deposition time (2 min), both the duration (Fig. 4f) and amount of drug release (Fig. 4e) increased with increasing nanosphere concentration. Our previous study demonstrated that the usage of pre-made *bm*SF nanospheres as coating building blocks instead of dissolved *bm*SF molecules remarkably increased the amount and duration of electropositive antibiotics (e.g. vancomycin) by establishing strong electrostatic and hydrophobic interactions between the drug and nanospheres [23]. Therefore, in the current study, *bm*SF nanospheres in the under-layer increased the loading capacity and reduced diffusion of encapsulated drugs as compared to their surrounding *bm*SF matrix. Consequently, the gradual increase in drug release duration and amount with increasing nanosphere concentration in *bm*SF_sol+sus_ was attributed to the higher content of nanospheres in under-layer architectures. Additionally, using the same nanosphere concentration (50%), the drug release dosage from the coating system increased with increased deposition time of the under-layer from 1 to 4 min (Fig. 4g).

**Fig. 5:**
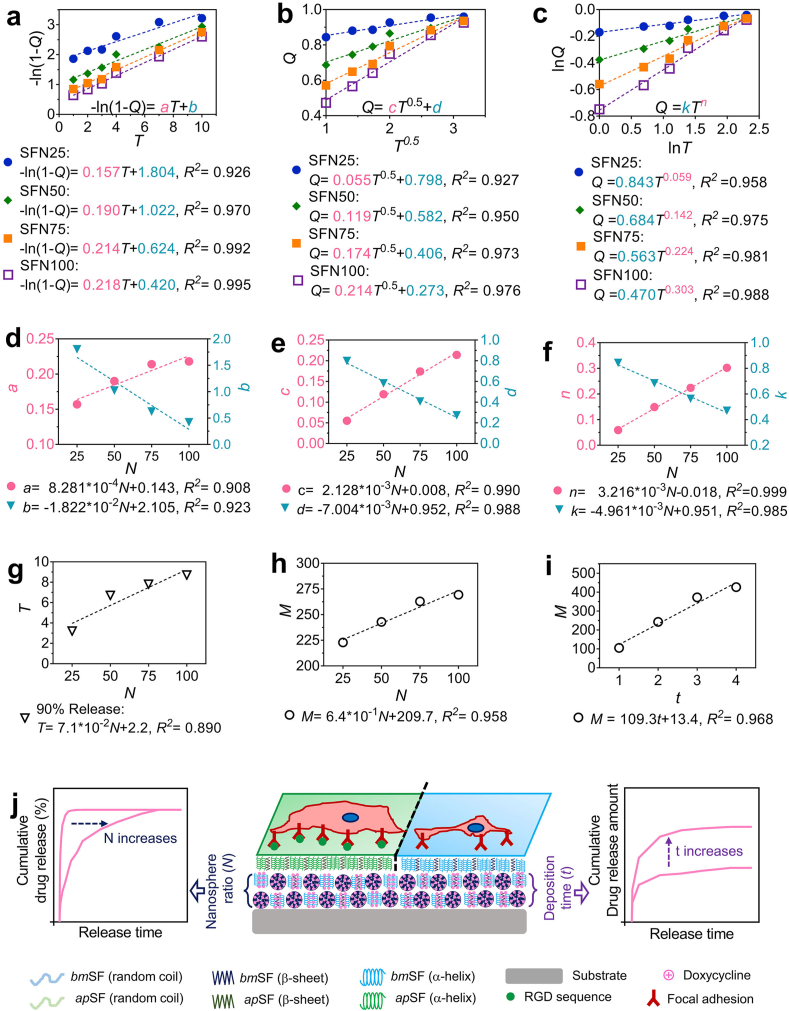
**Relationships between processing parameters and drug release kinetics of the coating system.** Release model fits of (**a, d**) first-order, **(b, e)** Higuchi, and **(c, f)** Korsmeyer-Peppas equations. Relationships between the constants in (**g**) first-order, **(h)** Higuchi, and **(i)** Korsmeyer-Peppas models and nanosphere concentration in EPD suspensions (*N*). **(g)** Relationship between the release time (90% maximum release) (i.e. “*T”*) and *N*. (**i)** Relationship between the maximum release amount (i.e. “*M”*) and *N* using the same under-layer deposition time (i.e. “*t”*) (2 min). (**h)** Relationship between *M* and *t* using the same *N* (50%). (**j**) Schematic illustrating that drug release duration and dosage of the coating system can be quantitatively tailored by manipulating processing parameters *N* and *t*. Moreover, the *ap*SF top-layer design enhances cytocompatibility of the coatings.

We further examined the relationship between processing parameters and drug release kinetics by fitting release data to three commonly used release models (i.e. the first-order, Higuchi, and Korsmeyer-Peppas) (Fig. 5a–c). The coefficients of determination (R^2^) of these fittings were all higher than 0.92, indicating that release data fitted well with all three models. The first-order and Higuchi model are diffusion-controlled release models [49], indicating that the release kinetics of coating system followed a diffusion-controlled manner. Moreover, the values of *n* in Korsmeyer-Peppas also indicate the specific drug release mechanisms [50]. With increase of nanosphere concentration from 25 to 100%, the values of *n* increased from 0.078 to 0.294. All values were below the crucial value 0.45, confirming that drug were released from the coating system according to a typical Fickian diffusion process [50]. The increase of *n* indicated that the increase of nanosphere content in the under-layer architecture delayed this diffusion process [50].

Furthermore, we found that all model constants displayed simple linear relationships (R^2^ = 0.90–0.99) with nanosphere concentration in *bm*SF_sol+sus_ (Fig. 5d–f), indicating that our coating system allows for prediction of release kinetics by tuning nanosphere concentrations. Additionally, a linear relationship (R^2^ = 0.89) was observed between nanosphere concentrations and release durations (90% maximum release) (Fig. 5g). This result confirms that the required drug release duration can be tuned for specific applications by adjusting the nanosphere concentration before coating preparation (Fig. 5j). On the other hand, the maximum drug release amount increased linearly with nanosphere concentration at the same deposition time (i.e. 2 min) (Fig. 5h), and was proportional to the under-layer deposition time using the same nanosphere concentration (i.e. SFN50) (Fig. 5i). This may be ascribed to the linear relationship between thickness of the under-layer and deposition time before reaching self-limitation (Fig. 1g). This result indicates that the deposition time can be used a tool to achieve the desired drug release dosage for a specific application (Fig. 5j).

Although promising results have been acquired only on using SF as coating matrix here, other commonly-used EPD coating matrix materials such as chitosan, alginate, and ceramics have also been widely used to assemble different nano- or micro-carriers for sustained therapeutic drug release [[51], [52], [53]]. Therefore, we envision that pre-defined architecture EPD strategy may be also be applied to those materials to achieve tunable drug delivery coating system. As for the developments of a medical applicable coating system, conversion to dosage forms, enhancement on drug loading efficiency, as well as pharmaceutical activity and release dynamics in different physiological stimuli (e.g., pH and anions) or *in vivo* conditions deserve further investigation.

## 4 Conclusions

A novel double-layered SF coating system was designed by sequential EPD of a mixture of both dissolved *bm*SF molecules and pre-made *bm*SF nanospheres at tunable ratios as under-layer covered by apSF molecules as top-layer to improve the control over drug delivery and the response of adherent cells. The model drug doxycycline was either pre-mixed with dissolved *bm*SF molecules or pre-loaded into pre-made *bm*SF nanospheres before deposition of the under-layers. The thickness and nanosphere content of the under-layers were controlled by the deposition time and nanosphere concentration in mixture precursors, respectively. The *ap*SF top-layer increased adhesion, spreading, and proliferation of fibroblasts as compared with *bm*SF top-layer and non-coated titanium substrates. Moreover, drug release amount and duration linearly increased with nanosphere concentration using the same deposition time. Furthermore, at a given nanosphere concentration, the increase of deposition time also enabled a linear enhancement on drug release amount. These results indicate that the drug release dosage and duration can by precisely controlled by altering two key processing parameters (nanosphere concentration in mixture precursors and deposition time). Overall, this study illustrates the strong potential of pre-defining the architecture of coatings to facilitate precise control over drug delivery.

## CRediT authorship contribution statement

**Xian Cheng:** Conceptualization, Data curation, Formal analysis, Visualization, Writing – original draft. **Dingpei Long:** Conceptualization, Data curation, Methodology. **Lili Chen:** Writing – review & editing. **John A. Jansen:** Writing – review & editing, Project administration. **Sander C.G. Leeuwenburgh:** Writing – review & editing, Project administration. **Fang Yang:** Conceptualization, Writing – review & editing, Project administration, Methodology.

## Declaration of competing interest

The authors declare that they have no known competing financial interests or personal relationships that could have appeared to influence the work reported in this paper.
