# Supplementary material for: Electrophoretic deposition of silk fibroin coatings with pre-defined architecture to facilitate precise control over drug delivery
Source: Bioact Mater. 2021 Apr 28;6(11):4243–54. doi: 10.1016/j.bioactmat.2021.03.046 (PMC8102429; doi:10.1016/j.bioactmat.2021.03.046)
Supplement: Multimedia component 1 [file mmc1.docx]

**Electrophoretic Deposition of Silk Fibroin Coatings with Pre-defined Architecture to Facilitate Precise Control Over Drug Delivery**

Xian Cheng^1,2^, Dingpei Long^3^, Lili Chen^4^, Sander C.G. Leeuwenburgh^1^, John A. Jansen^1^, and Fang Yang*

1. Department of Dentistry-Biomaterials, Radboud Institute for Molecular Life Sciences, Radboud University Medical Center, Philips van Leydenlaan 25, 6525 EX Nijmegen, The Netherlands.
2. Jiangsu Key Laboratory of Oral Diseases, Nanjing Medical University, Nanjing, 210029, P. R. China
3. Institute for Biomedical Sciences, Center for Diagnostics & Therapeutics, Georgia State University, Atlanta, GA 30302, USA
4. Department of Stomatology, Union Hospital, Tongji Medical College, Huazhong University of Science and Technology, Wuhan 430022, P. R. China

* Corresponding author E-mail: [Fang.Yang@radboudumc.nl](mailto:Fang.Yang@radboudumc.nl)

**Supplemental Figures:**


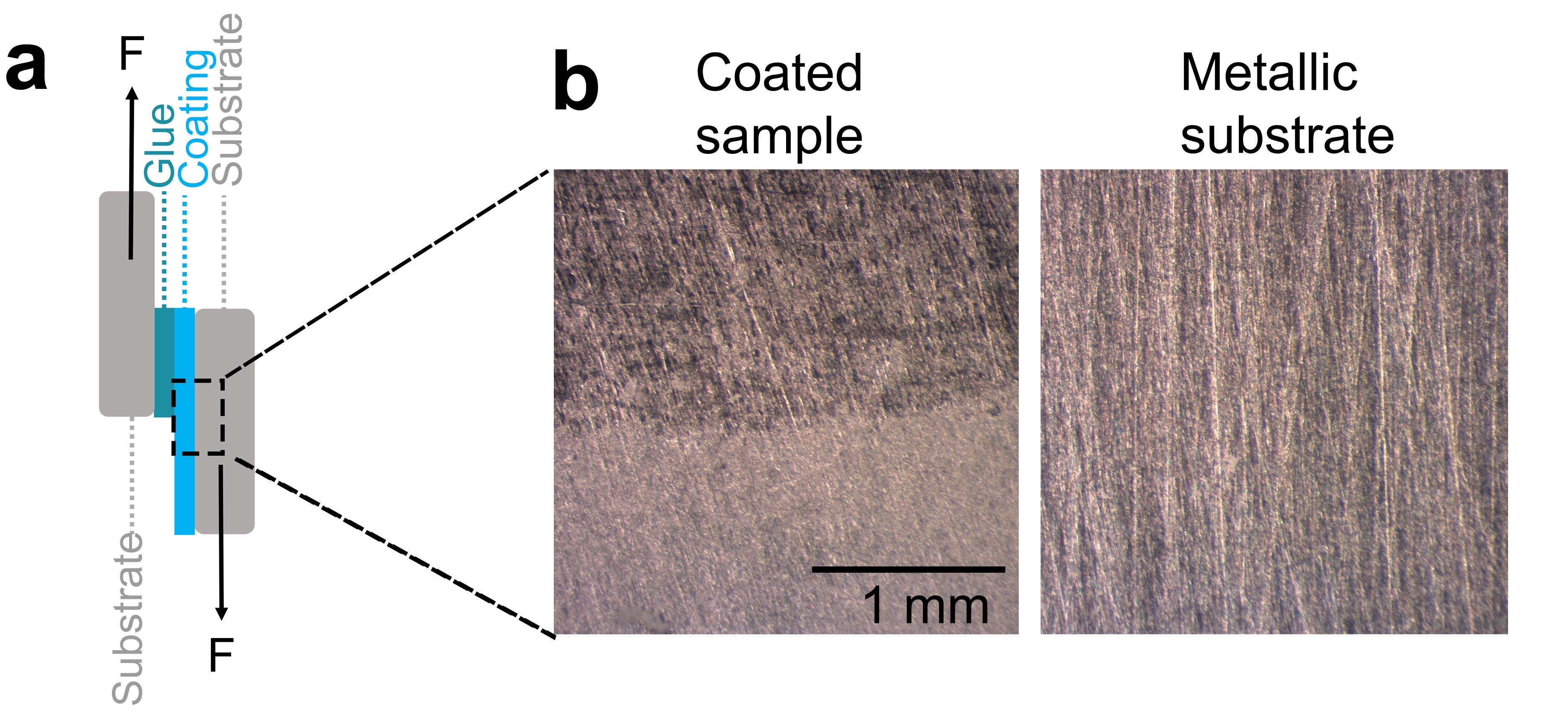


**Figure S1.** **Lap shear tensile test**. **(a)** Schematic diagram**. (b**) The exposure of substrates of coated samples (SFN50 used here as an example group) are checked using stereoscopic microscopy, ensuring that failure occurs at the interface between coating and substrates. The metallic substrate was applied as control.


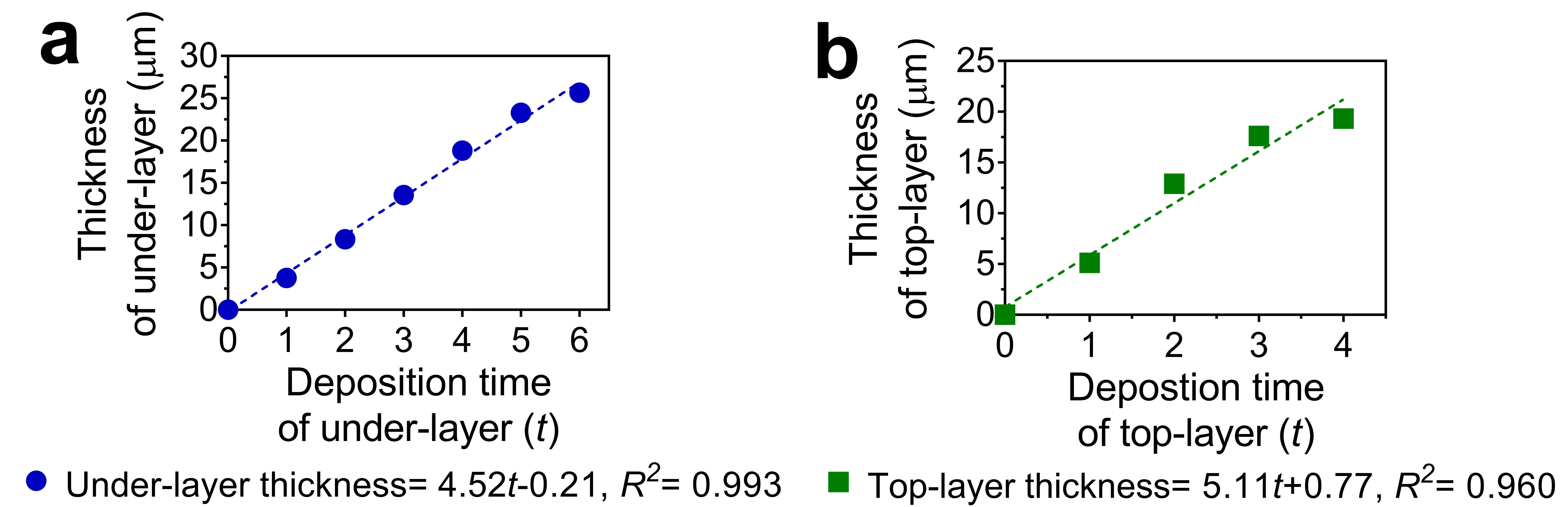


**Figure S2.** **Mathematical relationship** between deposition time and the thickness of **(a)** under-layer or **(b)** top-layer.


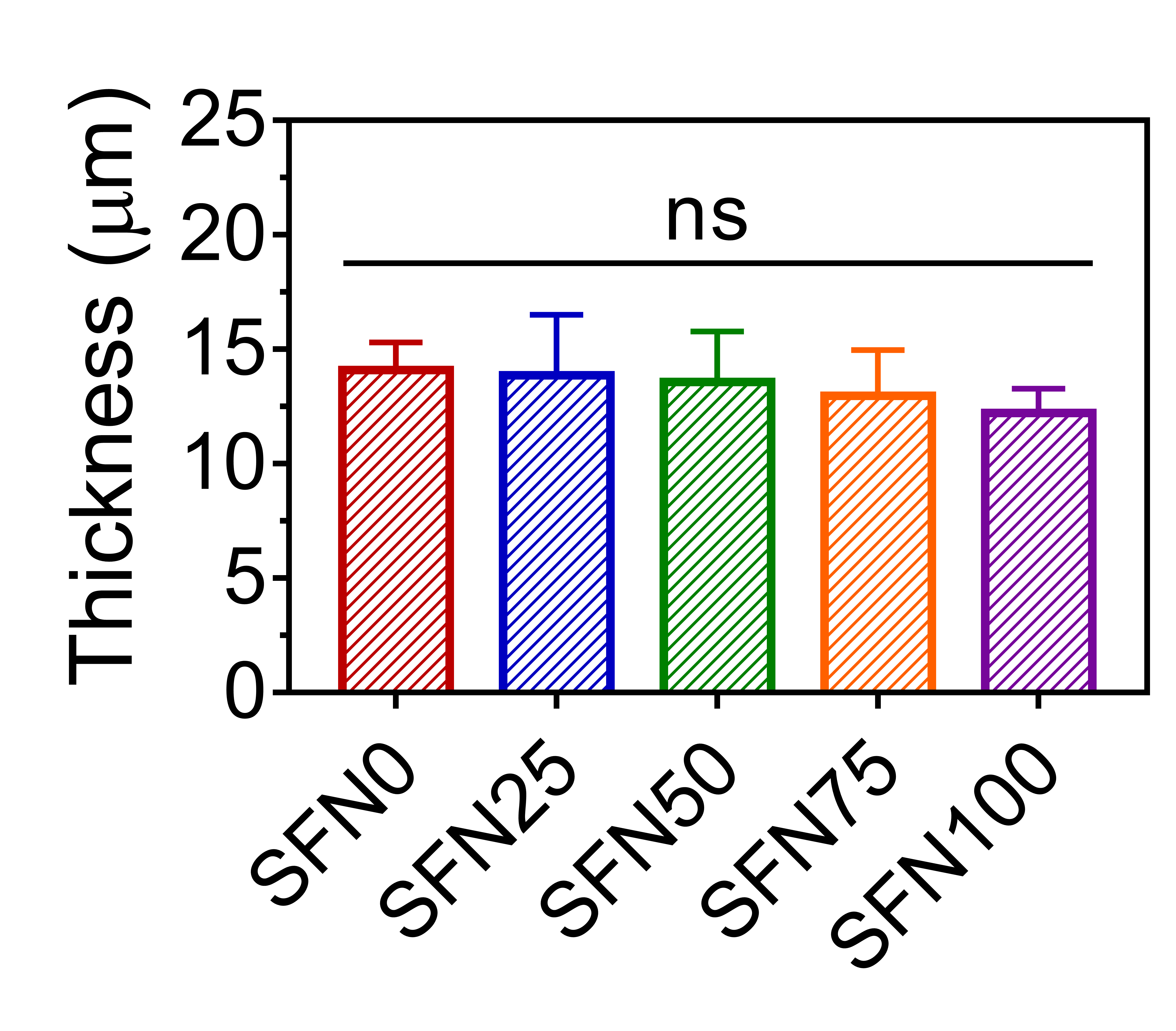


**Figure S3. Thickness of coatings.** Deposition time of the under-layer was 2 min and deposition time of the top-layer was 1min. Error bars represent one standard deviation. (ns: no significance, *: p < 0.05 and **: p < 0.01).


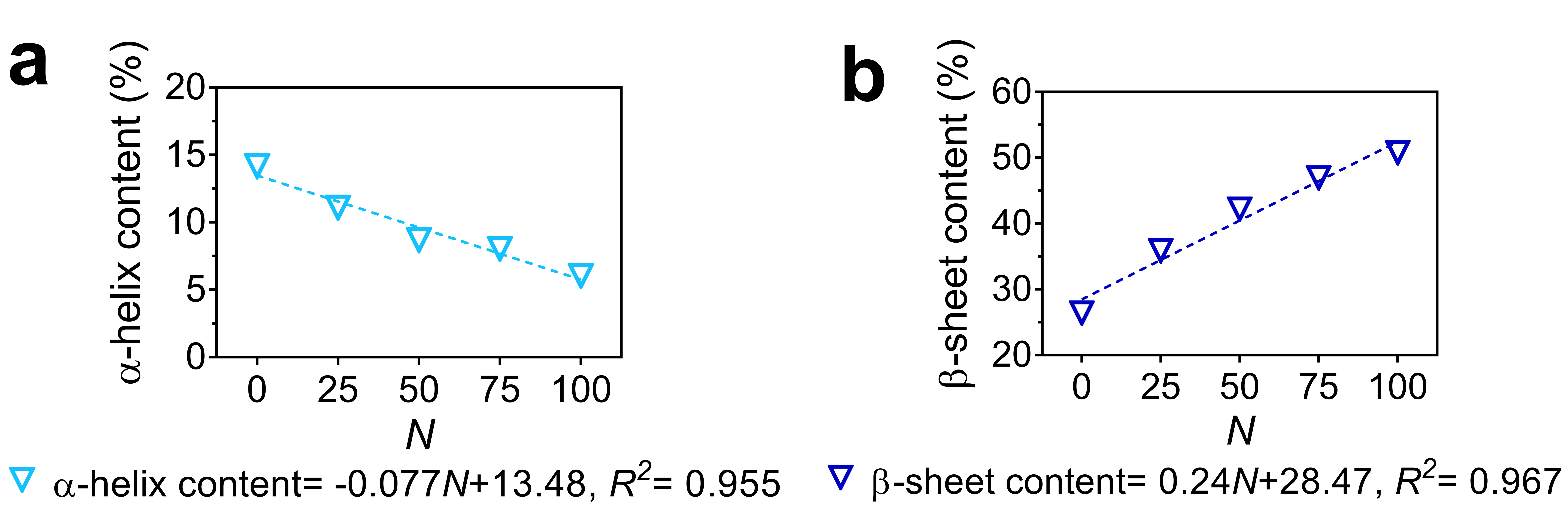


**Figure S4.** **Mathematical relationships** between processing parameter *N* and the content of **(a)** α-helix and (**b**) β-sheet conformation in underlayer.





**Figure S5.** **Surface characterizations of SFN50 and SFN50b.** **(a)** Surface topography observed by SEM. **(b)** Surface roughness of different coatings. **(c)** Surface wettability of different coatings measured by the water contact angle test, and representative images of water droplets on different material surfaces. Error bars represent one standard deviation. (ns: no significance, *: p < 0.05 and **: p < 0.01). Data in SFN50 group are obtained from Figure 2a, d, and e, which are displayed here for reasons of comparison.


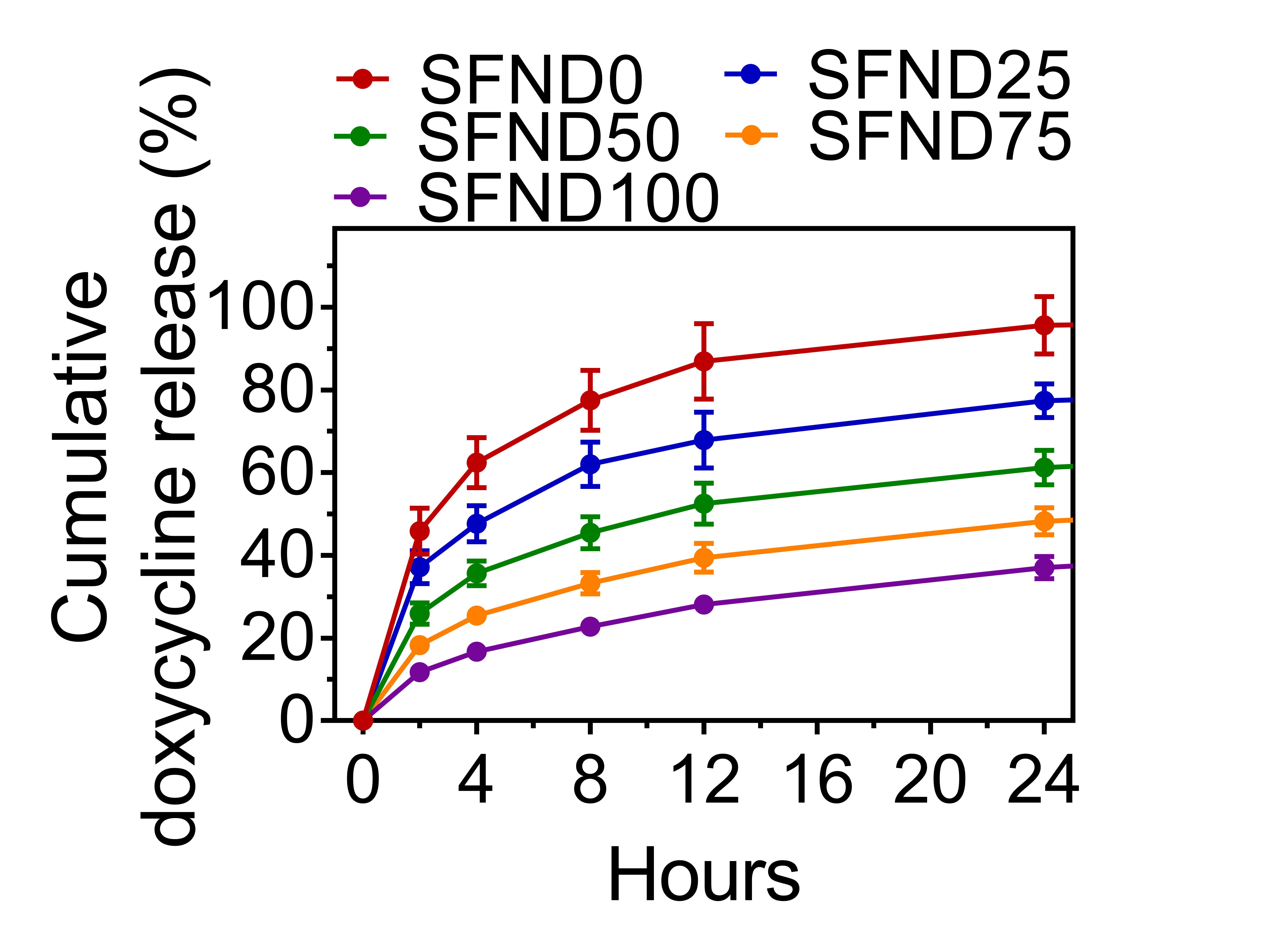


**Figure S6. Short-term drug release profiles** of the coating system. Error bars represent one standard deviation.


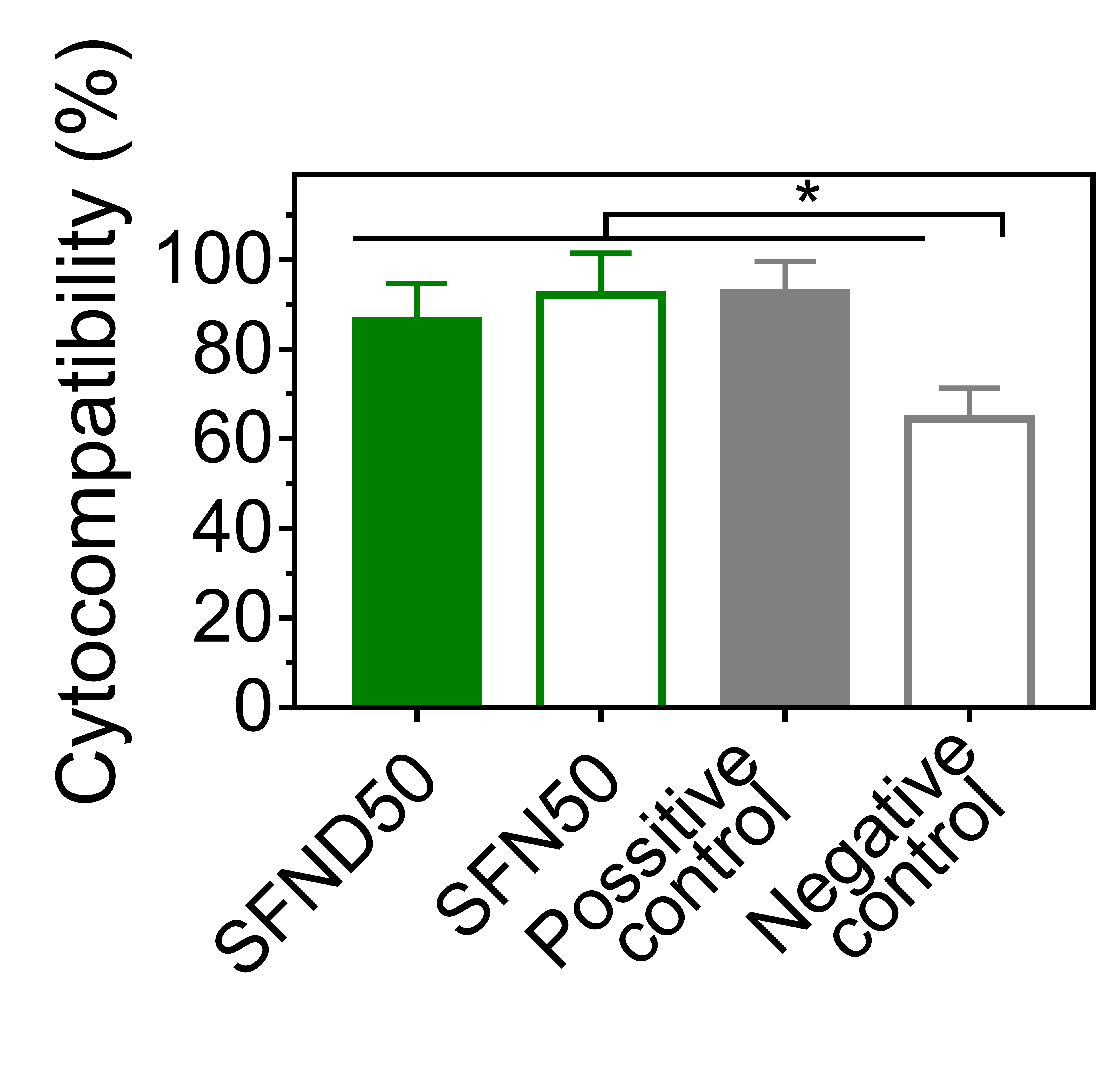


**Figure S7.** Cytocompatibility of SFND50 and SFN50 coatings.
